# Environmental and State-Level Regulatory Factors Affect the Incidence of Autism and Intellectual Disability

**DOI:** 10.1371/journal.pcbi.1003518

**Published:** 2014-03-13

**Authors:** Andrey Rzhetsky, Steven C. Bagley, Kanix Wang, Christopher S. Lyttle, Edwin H. Cook, Russ B. Altman, Robert D. Gibbons

**Affiliations:** 1 Department of Medicine, the University of Chicago, Chicago, Illinois, United States of America; 2 Institute for Genomics and Systems Biology, the University of Chicago, Chicago, Illinois, United States of America; 3 Computation Institute, Department of Human Genetics, the University of Chicago, Chicago, Illinois, United States of America; 4 Department of Genetics, Stanford University, Stanford, California, United States of America; 5 Committee on Genetics, Genomics, and Systems Biology, the University of Chicago, Chicago, Illinois, United States of America; 6 The Center for Health and the Social Sciences, the University of Chicago, Chicago, Illinois, United States of America; 7 Department of Psychiatry, Institute for Juvenile Research, University of Illinois at Chicago, Chicago, Illinois, United States of America; 8 Departments of Bioengineering, Genetics, Medicine, and Computer Science, Stanford University, Stanford, California, United States of America; 9 Departments of Medicine and Health Studies, the University of Chicago, Center for Health Statistics, Chicago, Illinois, United States of America; University of Florida, United States of America

## Abstract

Many factors affect the risks for neurodevelopmental maladies such as autism spectrum disorders (ASD) and intellectual disability (ID). To compare environmental, phenotypic, socioeconomic and state-policy factors in a unified geospatial framework, we analyzed the spatial incidence patterns of ASD and ID using an insurance claims dataset covering nearly one third of the US population. Following epidemiologic evidence, we used the rate of congenital malformations of the reproductive system as a surrogate for environmental exposure of parents to unmeasured developmental risk factors, including toxins. Adjusted for gender, ethnic, socioeconomic, and geopolitical factors, the ASD incidence rates were strongly linked to population-normalized rates of congenital malformations of the reproductive system in males (an increase in ASD incidence by 283% for every percent increase in incidence of malformations, 95% CI: [91%, 576%], *p*<6×10^−5^). Such congenital malformations were barely significant for ID (94% increase, 95% CI: [1%, 250%], *p* = 0.0384). Other congenital malformations in males (excluding those affecting the reproductive system) appeared to significantly affect both phenotypes: 31.8% ASD rate increase (CI: [12%, 52%], *p*<6×10^−5^), and 43% ID rate increase (CI: [23%, 67%], *p*<6×10^−5^). Furthermore, the state-mandated rigor of diagnosis of ASD by a pediatrician or clinician for consideration in the special education system was predictive of a considerable decrease in ASD and ID incidence rates (98.6%, CI: [28%, 99.99%], *p* = 0.02475 and 99% CI: [68%, 99.99%], *p* = 0.00637 respectively). Thus, the observed spatial variability of both ID and ASD rates is associated with environmental and state-level regulatory factors; the magnitude of influence of compound environmental predictors was approximately three times greater than that of state-level incentives. The estimated county-level random effects exhibited marked spatial clustering, strongly indicating existence of as yet unidentified localized factors driving apparent disease incidence. Finally, we found that the rates of ASD and ID at the county level were weakly but significantly correlated (Pearson product-moment correlation 0.0589, *p* = 0.00101), while for females the correlation was much stronger (0.197, *p*<2.26×10^−16^).

## Author Summary

Disease clusters are defined as geographically compact areas where a particular disease, such as a cancer, shows a significantly increased rate. It is presently unclear how common such clusters are for neurodevelopmental maladies, such as autism spectrum disorders (ASD) and intellectual disability (ID). In this study, examining data for one third of the whole US population, the authors show that (1) ASD and ID display strong clustering across US counties; (2) counties with high ASD rates also appear to have high ID rates, and (3) the spatial variation of both phenotypes appears to be driven by environmental, and, to a lesser extent, economic incentives at the state level.

## Introduction

Autism spectrum disorders (ASD) are a collection of chronic, complex neuropsychiatric diseases with well-characterized comorbidities and increasing apparent prevalence [1]. With few and limited effective treatments and considerable financial burden, its etiology remains a scientific puzzle. Evidence suggests that autism is highly heritable and clustered within families; consequently, much scientific attention has been dedicated to the discovery of predisposing genetic factors [2]–[7]. There is also evidence for environmental influences, such as prenatal exposure to pesticides or valproate, but it is challenging to account systematically for these factors because they are mostly undocumented. In addition, there are numerous factors that could affect or distort the observed variation in temporal and spatial disease prevalence: evolving diagnostic criteria, socioeconomic, legal, and cultural incentives for diagnosis [8], changing environmental exposures, and the accumulation of genetic burden in the growing human population. However, the relative importance of all these putative causal factors and confounders on ASD prevalence, the nature of interactions between contributing factors, and the underlying biological mechanisms, remain unclear.

Along these lines, geospatial clustering of ASD has been observed in California [9], [10], Texas [11], [12], North Carolina [13] and Utah [14]. Clustering could indicate the existence of localized risk factors, such as environmental toxins [15] or maternal education [13]. However, studies to date have focused primarily on within-state patterns and socioeconomic predictors, such as the level of parental education, the controversial financial incentives induced by state policies for special-education services, and broad environmental indicators. Now, the increasing availability of large administrative clinical datasets, with national coverage and fine spatial granularity, along with data on possible causal and confounding factors, provides an opportunity to compare the magnitudes of these and other factors within a unified mathematical framework.

Here, we report a mixed-effect Poisson regression analysis of the spatial incidence patterns of ASD and, for comparison, intellectual disability (ID). The data was derived from a very large insurance claims database containing nearly 100 million patients in the United States, which was augmented with census data to introduce additional county-level covariates that captured socioeconomic, demographic, and environmental effects. We present strong statistical evidence for environmental and legal factors driving the apparent spatial heterogeneity of both phenotypes, while documented socioeconomic factors and population structure have much weaker effects.

## Results

We analyzed the strength of disparate factors on the apparent incidence rates of ASD and ID by computationally interrogating insurance claims for approximately one third of the US population, using a bivariate-response, three-level, mixed-effects Poisson regression model with 50 free parameters, 44 of which correspond to the fixed effects of known factors while the remaining 6 account for the variance and covariance among the random effects (see Methods). The bivariate outcomes modeled by these parameters were the incidence counts for the ASD and ID phenotypes, tabulated separately for males and females in 3,111 counties, nested within 50 states (plus the District of Columbia) and adjusted for population size.

**Figure 1:**
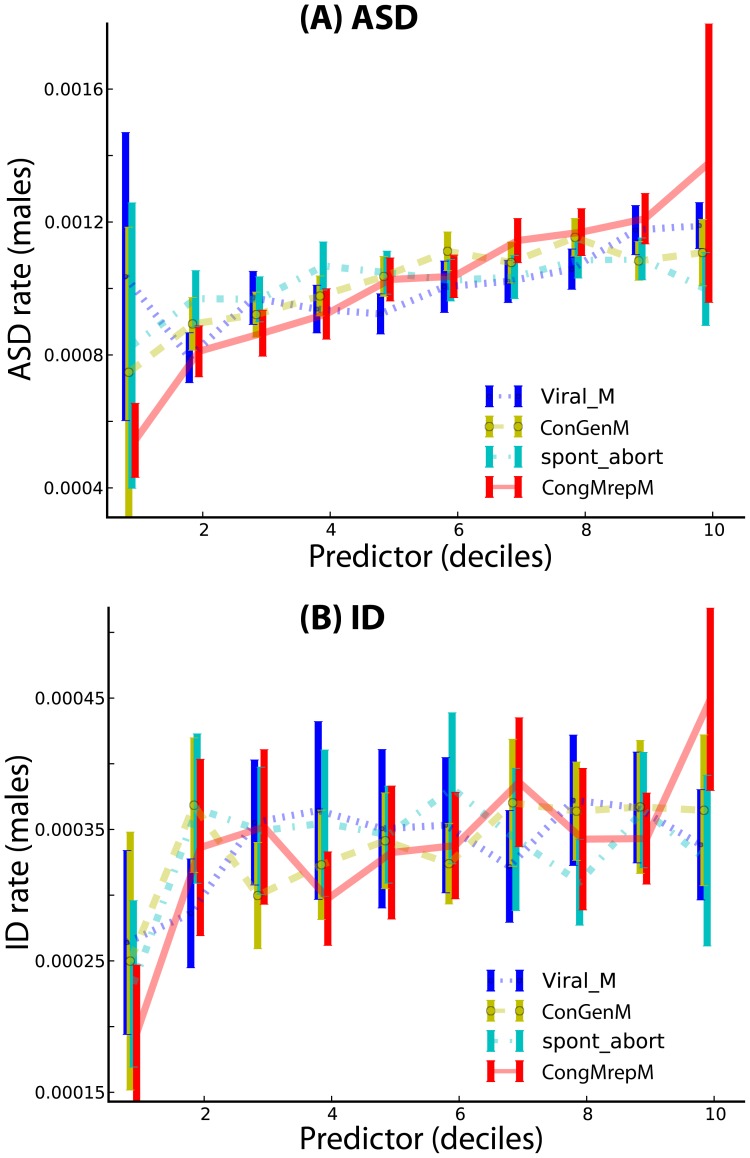
Deciles of putative predictors for ASD (A) and ID (B) per-male rates. Plots for females look essentially identical, but the absolute rate of incidence is lower (data not shown). The predictor variables shown here are *male congenital malformations of reproductive system* (*ConGenM*), viral infections in males of any age (*Viral_M*), congenital malformations excluding malformations of the genitals in males, *CongMrepM*, and spontaneous abortion (*spont_abort*).

**Figure 2:**
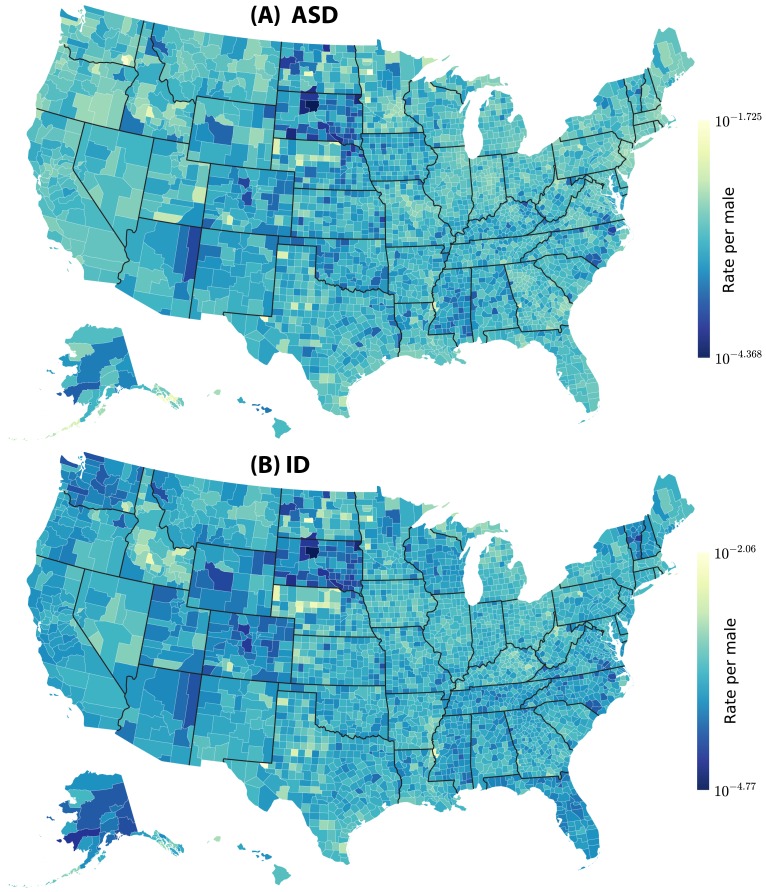
Estimated Poisson rates of incidence of ASD (A) and ID (B) per male individual of any age.

**Figure 3:**
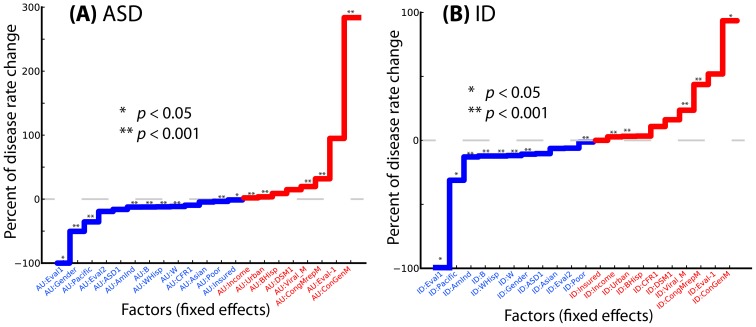
Comparison of fixed effects (geographically varying factors) governing rate variation in ASD (A) and ID (B). The asterisks indicate the level of significance of individual regression coefficients; see the figure key and Table 1.

**Figure 4:**
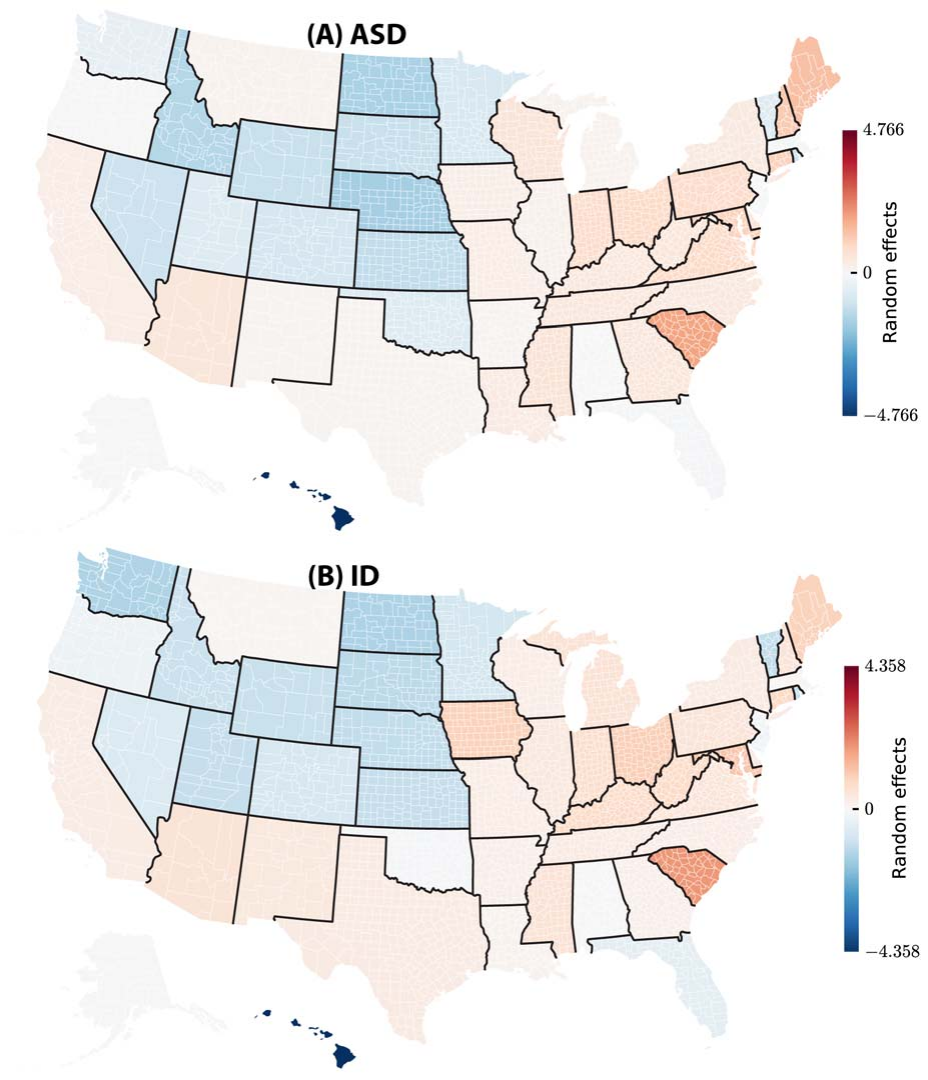
Total state-level random effects of ASD and ID incidence in the USA: (A) ASD and (B) ID. In the figures we color-coded the Empirical Bayes estimates of the state-level random effects, separately for ASD and ID. County- and state-level random effects model the unknown factors that vary geographically and govern differences in county-specific disease rates *after accounting for all fixed effects* (see Methods).

**Figure 5:**
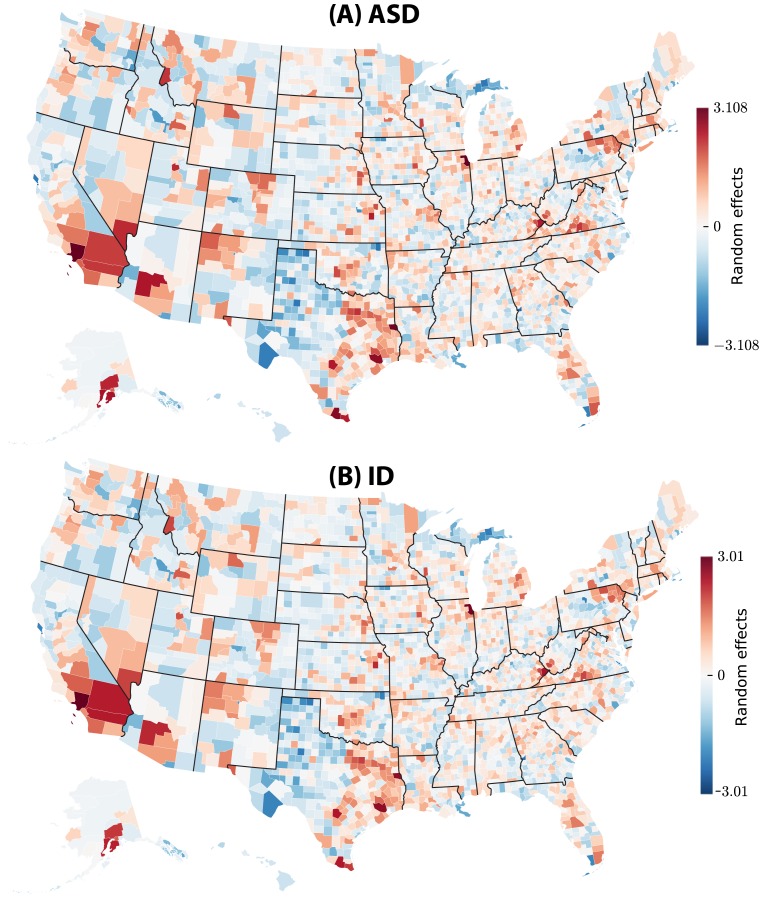
Total county-level random effects of ASD and ID incidence in the USA: (A) ASD and (B) ID. In the figures we color-coded the Empirical Bayes estimates of the state-level random effects, separately for ASD and ID. While county-specific random effects are directly comparable *within* the same state, comparison of these effects *across* different states is not meaningful, because each state-specific random effect determines the baseline disease rate for each county in the corresponding state, and these baseline rates vary across states.

The results are summarized in Figures 1–5 and Table 1: We observed clear spatial clusters for both ASD and ID. The raw data analyzed prior to complex modeling (see Figure 1) indicated that putative environmental variables were strongly predictive of rates of ASD and ID across the US. This trend persisted after the analysis was corrected for confounding variables using mixed-effect Poisson regression, see Figures 2–5. We found that ASD in males (normalized by county population) has a county-level mean rate of 0.1% per male of any age. The distribution of rates across counties is skewed: the median is 0.023% while maximum observed value is 5.2%. Similarly, for ID, the average rate over the whole country is 0.024% per any male, 0.025% per any female. The maximum per county per person rate reached 0.9% and 0.58% for males and females, respectively. Furthermore, for males, the rates of ASD and ID at the county level were weakly but significantly correlated (Pearson product-moment correlation 0.0589, *p* = 0.00101), while for females the correlation was much stronger (0.197, *p*<2.26×10^−16^). The estimated Poisson rates of incidence produced by model-based inference are shown in Figure 2. When direct comparison was possible, we compared our conclusions with those of prior studies ([9]–[14], see Table 2) and found that they were consistent.

**Table 1:** Markov chain Monte Carlo estimates of regression weights, corresponding event rate ratios (exponential of regression weights) and 95% event estimate credible intervals.

Parameter	Effect	Estimate	Event Rate	Estimate's CI		Rate's CI		*p*-value
				Lower	Upper	Lower	Upper	
AU		0.366869	1.443209	−0.972727	1.754813	0.378051	5.782366	0.56225
**AU:Eval1**	**−**	**−4.231486**	**0.014531**	**−8.877964**	**−0.328180**	**0.000139**	**0.720233**	**0.02475**
**AU:Gender**	**−**	**−0.699367**	**0.496900**	**−0.709451**	**−0.689520**	**0.491914**	**0.501817**	**<6×10^−5^**
**AU:Pacific**	**−**	**−0.438859**	**0.644772**	**−0.700535**	**−0.173497**	**0.496320**	**0.840720**	**0.00150**
AU:Eval2	−	−0.213462	0.807783	−2.921955	2.470602	0.053828	11.829566	0.88300
AU:ASD1	−	−0.175057	0.839409	−1.582356	1.164197	0.205490	3.203350	0.83788
**AU:AmInd**	**−**	**−0.131310**	**0.876946**	**−0.224315**	**−0.054407**	**0.799063**	**0.947047**	**<6×10^−5^**
**AU:B**	**−**	**−0.130750**	**0.877437**	**−0.216518**	**−0.053650**	**0.805318**	**0.947764**	**<6×10^−5^**
**AU:WHisp**	**−**	**−0.127715**	**0.880104**	**−0.213386**	**−0.052847**	**0.807844**	**0.948525**	**<6×10^−5^**
**AU:W**	**−**	**−0.121627**	**0.885479**	**−0.210127**	**−0.046265**	**0.810481**	**0.954789**	**<6×10^−5^**
AU:CFR1	−	−0.099843	0.904979	−1.789320	1.490554	0.167074	4.439554	0.95962
AU:Asian	−	−0.045453	0.955565	−0.136669	0.036981	0.872259	1.037673	0.34550
**AU:Poor**	**−**	**−0.035009**	**0.965597**	**−0.049626**	**−0.020552**	**0.951585**	**0.979658**	**<6×10^−5^**
**AU:Insured**	**−**	**−0.012102**	**0.987971**	**−0.021311**	**−0.003377**	**0.978914**	**0.996629**	**0.00725**
**AU:Income**	**+**	**0.032462**	**1.032995**	**0.023206**	**0.041997**	**1.023477**	**1.042891**	**<6×10^−5^**
**AU:Urban**	**+**	**0.036144**	**1.036805**	**0.034184**	**0.038026**	**1.034775**	**1.038758**	**<6×10^−5^**
AU:BHisp	+	0.085030	1.088750	−0.101763	0.254757	0.903244	1.290148	0.34987
AU:DSM1	+	0.138073	1.148059	−1.120050	1.462245	0.326263	4.315637	0.80438
**AU:Viral_M**	**+**	**0.180043**	**1.197269**	**0.122006**	**0.238505**	**1.129761**	**1.269350**	**<6×10^−5^**
**AU:CongMrepM**	**+**	**0.276762**	**1.318852**	**0.119429**	**0.421873**	**1.126853**	**1.524815**	**0.00025**
AU:Eval-1	+	0.667525	1.949407	−0.773906	2.342192	0.461208	10.404017	0.36600
**AU:ConGenM**	**+**	**1.344962**	**3.838041**	**0.752946**	**1.912499**	**2.123246**	**6.769986**	**<6×10^−5^**
ID		−0.227338	0.796652	−1.393526	0.980272	0.248199	2.665181	0.66487
**ID:Eval1**	**−**	**−4.535572**	**0.010721**	**−8.642693**	**−1.122879**	**0.000176**	**0.325342**	**0.00637**
**ID:Pacific**	**−**	**−0.373752**	**0.688148**	**−0.611520**	**−0.120910**	**0.542526**	**0.886114**	**0.00487**
**ID:AmInd**	**−**	**−0.138735**	**0.870459**	**−0.216428**	**−0.069898**	**0.805391**	**0.932489**	**<6×10^−5^**
**ID:B**	**−**	**−0.130235**	**0.877889**	**−0.205861**	**−0.066371**	**0.813946**	**0.935784**	**<6×10^−5^**
**ID:WHisp**	**−**	**−0.130204**	**0.877916**	**−0.203526**	**−0.066016**	**0.815849**	**0.936116**	**<6×10^−5^**
**ID:W**	**−**	**−0.126588**	**0.881097**	**−0.200798**	**−0.060878**	**0.818078**	**0.940938**	**<6×10^−5^**
**ID:Gender**	**−**	**−0.114004**	**0.892254**	**−0.127122**	**−0.101693**	**0.880626**	**0.903307**	**<6×10^−5^**
ID:ASD1	−	−0.109237	0.896518	−1.370748	1.037993	0.253917	2.823544	0.89650
ID:Asian	−	−0.065537	0.936564	−0.146325	0.003463	0.863877	1.003469	0.05613
ID:Eval2	−	−0.063085	0.938864	−2.418593	2.326817	0.089047	10.245279	0.97462
ID:Poor	−	−0.013068	0.987017	−0.027450	0.001640	0.972923	1.001641	0.08038
ID:Insured	+	0.004845	1.004857	−0.004074	0.014573	0.995934	1.014680	0.30088
**ID:Income**	**+**	**0.027171**	**1.027543**	**0.018098**	**0.036522**	**1.018263**	**1.037197**	**<6×10^−5^**
**ID:Urban**	**+**	**0.031155**	**1.031645**	**0.029295**	**0.032969**	**1.029728**	**1.033518**	**<6×10^−5^**
ID:BHisp	+	0.032889	1.033436	−0.129128	0.194847	0.878861	1.215125	0.66837
ID:CFR1	+	0.102626	1.108077	−1.421645	1.452865	0.241317	4.275346	0.82900
ID:DSM1	+	0.149366	1.161098	−0.971742	1.285555	0.378423	3.616675	0.76525
**ID:Viral_M**	**+**	**0.211402**	**1.235409**	**0.153162**	**0.269626**	**1.165514**	**1.309475**	**<6×10^−5^**
**ID:CongMrepM**	**+**	**0.362239**	**1.436542**	**0.210009**	**0.516094**	**1.233689**	**1.675470**	**<6×10−5**
ID:Eval-1	+	0.417890	1.518754	−0.871996	1.849778	0.418116	6.358408	0.54113
**ID:ConGenM**	**+**	**0.660186**	**1.935152**	**0.013537**	**1.255318**	**1.013629**	**3.508954**	**0.03838**

The 3-level Poisson mixed-effect model included 12,444 level-1 units (incidence counts for two diseases), 3,111 Level-2 units (counties), and 51 level-3 units (states). The table is designed to mirror Figure 1: fixed-effect parameter estimates for autism spectrum disorders (AU) are followed by the corresponding estimates for intellectual disability (ID). For each group of diseases the first parameter listed (AU and ID, respectively) is the intercept. The rest of the fixed-effect parameters are ordered from the strongest negative effect to the strongest positive effect, as in Figure 1. **Abbreviations:** AU – autism spectrum disorders; ID – intellectual disabilities; AmInd – proportion of American Indians; Asian – proportion of Asians; WHisp – White Hispanics; W – White non-Hispanic; BHisp – black Hispnics; B – black non-Hispaic; Pacific – Pacific Islanders; Insured – proportion of insured; Poor – proportion of poor; Urban – proportion of urban; CongMrepM – congenital malformations excluding malformations of genitals in males; ConGenM – congenital malformations of genitals in males; Viral_M – viral infections affecting males; ASD – inclusion of Autism Spectrum Disorders; CFR – Code of Federal Regulations; DSM – requirement of reference to Diagnostic and Statistical Manual of Mental Disorders; Eval – rigor of evaluation of diagnosis veracity. The regulations were encoded in the following way (23). For CFR: Code −1 if criteria included information from the autism section of CFR only, and +1 if the criteria incorporated additional non-CFR information. For DSM: Code −1 if the entire DSM-IV-TR criteria were used, and +1 otherwise. For ASD: −1 if autism spectrum disorders were included in diagnostic criteria, and +1 if they were not included. For Eval: −1 if a diagnosis by a pediatrician or clinician was mandatory, 1 if a diagnosis of autism or autism spectrum disorders by a pediatrician or clinician was mandated, and 2 if no requirements in addition to those mandated by the Individuals with Disabilities Education Act were added.

**Table 2:** Markov chain Monte Carlo estimates of covariances and correlations of random effects across two phenotypes.

	**Covariance**	**Correlation**	**Cov: 95% CI**	
			**Lower**	**Upper**
AU:AU (State)	3.126	1	0.6062	7.239
AU:ID (State)	2.666	0.974	0.5198	6.088
ID:ID (State)	2.398	1	0.5610	5.358
AU:AU (County)	0.7699	1	0.7142	0.8285
AU:ID (County)	0.6960	0.962	0.6462	0.7488
ID:ID (County)	0.6796	1	0.6261	0.7357

Accumulating evidence [16]–[28] suggests that the rate of birth malformations, especially of those affecting the reproductive system in newborn boys [17], adjusted for population size and structure, could serve as an indicator of average parental exposure to toxins within a geographic unit. After controlling for ethnicity, gender, and socioeconomic factors, the strongest predictor of ASD was the rate of male congenital malformations of the reproductive system, used as an approximate measurement for exposure to teratogens, based on extensive epidemiological evidence ([16], [17], see Figure 3 and Discussion). Every additional percent incidence of male congenital malformations of the reproductive system was predictive of a 283% increase in the rate of the ASD incidence (95% confidence interval, CI: [91%, 576%], *p*<6×10^−5^). Similarly, non-reproductive congenital male malformations accounted for a 31.8% ASD rate increase (CI: [12%, 52%], *p*<6×10^−5^). In contrast, male congenital malformations of the reproductive system were barely significantly predictive for ID (94%, CI: [1%, 250%], *p* = 0.0383). However, the effect of non-reproductive congenital malformations in males on ID incidence was statistically significant and strong: an increase of 43% (CI: [23%, 67%], *p*<6×10^−5^). Another variable significantly affecting both ASD and ID was population-adjusted incidence of viral infections in males (Table 1, Figure 3, and Discussion). Moreover, comorbidity analysis demonstrated that male children with ASD are 5.53 times more likely to have congenital genital malformations than unaffected males (odds ratio 95% CI [5.22, 5.87], *p*<2.2×10^−16^, Fisher's exact test).

Male congenital malformations of the reproductive system are subdivided in the ICD9 taxonomy [29], which was used to encode the data in this analysis, into unspecified malformations, hypospadias (abnormally placed external urethral orifice), epispadias (the urethra does not develop into a full tube), micropenis, congenital chordees and undescended testicles. The US average incidence rate for all male congenital malformations of reproductive system was 0.2687% per male of any age group; of these 18% were hypospadias (0.049% rate), 6% congenital chordees (0.0161% rate), 1% micropenis (0.00275% rate), and 0.083% epispadias (0.00227% rate). The rest of the malformations were in the unspecified category; undescended testicles were not encoded explicitly. Per-county rates of hypospadias and congenital chordees were significantly correlated with each other (Pearson's *r* = 0.34, 95% CI [0.31, 0.372], *p*<2.2×10^−16^), as were hypospadias and epispadias (*r* = 0.066, 95% CI [0.031, 0.101], *p* = 0.00022). The highest per county rates of malformations were 2.4% (all male genital malformations), 1.1% (hypospadias), 0.91% (chordees), 1.1% (epispadias), and 0.23% (micropenis). There were counties with no reported malformations (zero apparent rate). All discussed groups of malformations were significantly correlated with autism rates. Female birth malformations of reproductive system, variable *ConGenF*, showed very similar disease-specific predictive behavior to the congenital male malformations of reproductive system, variable *ConGenM*. The female malformations were predictive of an increase in both ASD and ID, but the magnitude of the statistical effect associated with this factor was much smaller than *ConGenM*, although highly correlated.

Both ASD and ID showed significant gender-specific incidence effects, with males affected more frequently than females; this was more extreme for ASD (Table 1 and Figure 3). Using ethnicity variables to account for genetic heterogeneity of the US population, corrected for socioeconomic factors, such as the mean county-specific income, we found the incidence of the two diseases significantly varied across ethnic groups (Table 1), with Pacific Islanders, for example, having significantly lower risk for both diseases. The per capita income of the county was weakly positively correlated with the incidence rates for both diseases: the income variable was associated with 3.2% rate increase per every additional $1,000 of income above the country average for ASD (CI: [2.3%, 4.2%], *p*<6×10^−5^) and a 2.7% rate increase for ID (CI: [1.8%, 3.7%], *p*<6×10^−5^). Other important socioeconomic predictors included the percentage of urban population in a county; a one percent increase in urbanization predicted about a 3% increase in ASD and ID incidence (Table 1). Our analysis also indicated that state-specific laws [30], [31] had a large but only marginally significant effect on the incidence rates of ASD and ID (Table 1, Figure 1). The strictest form of diagnostic evaluation (variable *Eval*, the state-mandated diagnosis of autism or autism spectrum disorders by a pediatrician or clinician for consideration in the special education system) was predictive of a considerable decrease in ASD and ID incidence rates, 98.6% (CI: [28%, 99.99%], *p* = 0.02475) and 99% (CI: [68%, 99.99%], *p* = 0.00637) respectively.

## Discussion

By analyzing the spatial incidence patterns of autism and intellectual disability drawn from insurance claims for nearly one third of the total US population, we found strong statistical evidence that environmental factors drive the apparent spatial heterogeneity of both phenotypes while economic incentives and population structure appear to have relatively large albeit weaker effects. The strongest predictors for autism were associated with the environment: congenital malformations of the reproductive system in males (an increase in ASD incidence by 283% for every per cent of increase in the incidence of malformations), non-reproductive congenital malformations (31.8% ASD rate increase), and viral infections in males (19% ASD rate increase). For ID we observed similar but weaker effects: 93% increase of ID rate for every per cent of increase in congenital malformations of the reproductive system in males, 43% increase for per cent of non-reproductive congenital malformations, and 23% for viral infections in males.

We highlight the role of male congenital genitourinary malformations as an approximate measurement of environmental exposure to unmeasured developmental risk factors, including toxins. Some infants are born with congenital malformations with unknown genetic etiology–not explained by known Mendelian mutations or detectable chromosomal aberrations. At least a fraction of such birth defects may be due to parental exposure to environmental insults. The environmental factors implicated so far include pesticides [18], [19], environmental lead [20], sex hormone analogs [21], [22], medications [23], plasticizers [24], and other synthetic molecules [25]. More generally, the risk of congenital birth defects is statistically linked to parental occupation [26]–[28]. There is a statistically significant increase in birth defects associated with some maternal occupations (janitor, maid, landscaper), and significant decrease associated with others (non-preschool teacher) [27], [28]. It is very likely that the list of environmental factors potentially affecting development of human embryo is large and yet predominantly undocumented; correspondingly, detailed statistics on these factors do not exist.

It is known that some birth malformations are caused by *de novo* genetic events, such as large copy number variants that have been found to increase the risk for ASD by approximately 400% [32]. Single-gene deletions, for example, involving CHD7 are known to cause CHARGE syndrome [33], [34] associated with genital abnormalities and putatively associated with ASD [35]. However, these genetic events may have currently poorly identified environmental triggers, and 70 to 80% of male congenital malformations of the reproductive system have no clear genetic causes [36]. Instead, they appear to be driven by specific environmental insults that were not serious enough to lead to more serious adverse events during pregnancy, such as spontaneous abortion. Therefore, in this study, we used the rate of birth malformations as a surrogate measure for environmental burden.

The hypospadias of the male urethra can arise during early embryonic development, specifically weeks 9–12 (p. 206 in [36]). This window corresponds to the time when cell division and migration takes place in brain development. Furthermore, maternal exposure to estrogen and estrogen analogs in animal models affects both brain and genital development in male progeny (p. 206 in [36]), and small physical malformations appear enriched in autistic children compared to healthy children [37].

Following similar logic, in addition to causing birth defects, environmental toxins, such as pesticides [38], [39] can substantially weaken the human immune system, especially in men, which results in more frequent infections. (The rates of female viral infections were highly correlated with male viral infections; these can serve a somewhat weaker fixed effect predictor, data not shown.) This suggests that per capita rate of viral infection, when socioeconomic and other biological factors have been controlled for, may serve as another environmental indicator, although specifics of the causal, biological mechanisms remain unresolved. In our analysis we found that the rate of viral infection in males was significant for both ASD and ID, see Table 1.

Importantly, the effect of state-level regulations involving ASD appeared relatively large in magnitude (over 98% ASD and ID rate decrease) but with a wide confidence interval and inconsistent effects across states, resulting in only marginal significance. Furthermore, our estimates of random effects at the state and county levels, see Figure 2, suggest that additional yet unknown confounder factors exist at both state and county levels, as is evident from the clear state boundaries seen in Figure 2.

As with other statistical analyses (see Table 3), significant associations are not necessarily causal. Identified predictor variables may reflect underlying mechanisms, or may be correlated with unmeasured causal factors. However, we have included variables, such as the rate of birth malformations and male viral infections, that have well-documented environmental causes. Overall, this increases our confidence in their scientific relevance. Furthermore, because we have controlled for many county-level socioeconomic variables, strong state-specific effects are almost certainly rooted in legal and regulatory differences that exist at this level.

**Table 3:** Summary of the prior state-level studies regarding geographic clustering of ASD.

	Study (state)				
	[19] (CA)	[20] (CA)	[21], [22] (TX)	[23] (NC)	[24] (UT)
**Data source**	All live births and diagnostic records for children born in CA between 1992 and 2000.	All live births in CA occurring in 1996–2000	Administrative educational data for prevalence of autism and other special education categories for the academic years 2000–2001 through 2005–2006.	Record-based surveillance for 8 NC counties biennially from the Autism and Developmental Disabilities Monitoring Network.	Record-based surveillance for eight-year-old children born in 1994 and living in Utah in 2002 from the Utah Registry of Autism and Developmental Disabilities Program.
***Cases + controls***	4,906,926	2,453,717	4,057,712	11,034	26,108
***Cases***	18,731	9,900	7,022 (ASD+ID)	532 (ASD), 1,028 (ID)	99 (ASD-only), 33 (ASD and ID), 113 (ID-only)
***Modeling formalism***	Multilevel logistic regression.	Spatial clustering and bivariate mixed Poisson regression.	Multilevel Poisson regression.	Generalized additive model.	Multiple single-variable logistic regressions.

Our results have implications for the ongoing scientific quest for the etiology of neurodevelopmental disorders. We provide evidence that routinely expanding the scope of inquiry to include environmental, demographic and socioeconomic factors, and governmental policies at a broad scale in a unified geospatial framework. It appears that detailed documentation of environmental factors should be recorded and used in genetic analyses of ASDs and failure to do so risks omitting important information about possibly strong confounders.

## Materials and Methods

### Ethics statement

Our analysis involved de-identified patient data and was approved by the University of Chicago Institutional Review Board.

Our multi-level, mixed-effects model predicted the incidence of ASD and ID conditional on several individual-level, county-level, and state-level covariates. For the regression analysis described below, we used county level variables to predict disease rate. In the analysis of the comorbidity between congenital malformations and ASD or ID, we used patient-level data.

### Data

We used the Truven Health Analytics MarketScan Commercial Claims and Encounters Database to provide geocoded diagnosis counts by gender. This database spans the years 2003 to 2010 and consists of approved commercial health insurance claims for between 17.5 and 45.2 million people annually, with linkage across years, yielding a total of approximately 105 million patient records. (Note that, consistent with low prevalence of both phenotypes, only a small proportion of individuals described in this enormous dataset were diagnosed with either ASD or ID.) This national database contains information contributed by well over 100 insurance carriers and large self-insuring companies. We scanned approximately 4.6 billion inpatient and outpatient service claims and identified almost 6 billion diagnostic codes. After removing duplicates, almost 1.3 billion diagnostic codes were found to be associated with over 99.1 million individuals, yielding approximately 12.89 unique diagnostic codes per individual. Claims were de-identified, that is, all patient-level personal information was redacted, and geocoded at the county level by Truven, and thus, this did not require any additional processing on the authors' part.

The MarketScan insurance claims dataset is not a random sample of the USA population. This is because compilation of this dataset required reaching agreements between Truven and numerous individual insurance providers to share data, and the insurance providers inherently had uneven and non-random coverage of geographic areas. It is possible, therefore, that the dataset carries traces of hidden correlations imposed by the data collection method. Furthermore, while the entire USA is well represented in the data, it is possible that coverage across geographic areas is not perfectly proportional to population density.

### Statistical analysis

We framed our analysis as a mixed-effect regression model for Poisson-distributed count data [40], independently implemented in SuperMix [41], lme4 [42], [43], MCMCglmm [44], and GLLAMM [45], [46]. The choice of the Poisson model was motivated by the countable nature of data and the rarity of the disease incidence events.

The model parameters were estimated using a joint statistical inference as follows. Most of the parameters (44 out of 50) were real-valued coefficients representing regression weights of individual factors, such as average income in county, percentage of ethnic groups, per cent of urban and poor population, see Table 1. The factors that are *a priori* suspected to be relevant to disease incidence and are deliberately included into the model, are referred to in the mixed effect model formalism as the *fixed effects*. In addition, the model included zero-centered and normally distributed *random effects*, uncorrelated for the same disease between a county and the encapsulating state, but geographically correlated between two diseases, see equations below and Figures 4 and 5. The six parameters associated with the random effects included the variances and covariances for the state- and county-level random effects (see Table 2 and equations below). Note that the random effects themselves were not parameters, but Gaussian zero-centered random variables.

Fixed effects by design have stochastic but predictable influence on data, while random effects describe zero-centered random influence, not captured by the fixed effects. Below we present more formal formulation of the model.

### Fixed-effect variables

The Truven database was augmented with US census data [30] consisting of county-level measurements for a variety of socioeconomic, demographic, and geospatial factors. Our fixed-effect county-level covariates were gender, *Gender*, average per capita income, *Income*, percent ethnicity (separately for American Indians, *AmInd*, Asians, *Asian*, White Hispanics, *WHisp*, White non-Hispanics, *W*, Black Hispanics, *BHisp*, Black non-Hispanics, *B*, and Pacific Islanders, *Pacific*), and the proportions of various socioeconomic groups (poor, *Poor*, urban, *Urban*, insured, *Insured*). Our county-level environmental indicators used as fixed-effect covariates (normalized by county population size) comprised congenital malformations excluding malformations of the genitals (separately for females and males, *CongMrepF* and *CongMrepM*, respectively), congenital malformations of the genitals (separately for females and males, *ConGenF* and *ConGenM*, respectively), viral infections (separately for females and males, *Viral_F* and *Viral_M*, respectively), ectopic pregnancy (*ect_pr*), abnormal conception (*abnormal_concept*), spontaneous abortion(*spont_abort*), and multiple gestations (*mult_gest*). The county-level environmental indicators were extracted from the Truven database and normalized by county population, separately for males and females.

To account for variation in policies for special education eligibility and reimbursement, we used four variables derived by hand-coding the state policies for eligibility in special education programs under the Individuals with Disabilities Education Act [31] with categorical variables: (i) *CFR*, to indicate whether state criteria met (−1) or, alternatively, exceeded Code of Federal Regulation requirements (1), (ii) *DSM*, to indicate whether state criteria mentioned all of the criteria from the Diagnostic and Statistical Manual of Mental Disorders (−1, if no, and 1, if yes), (iii) *ASD*, to indicate whether Autism Spectrum Disorder criteria were mentioned in the state criteria (−1 if no, 1 if yes), and (iv) *Eval*, to indicate the degree of diagnostic rigor required by the state (−1, −2, 1, 2). Details of the coding are described in the abbreviations. All predictor variables were mean-centered.

### Assumptions of the model

The assumptions of the Poisson regression model [40] were as follows. First, we assumed that the data, corresponding to the observed counts of people within each county diagnosed with either ASD or ID, were generated by a Poisson process, with rate (λ*_ijkl_*) varying over counties,
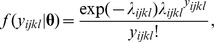
where: θ is a vector of all 50 model parameters, (**b**, **Σ**). The observed counts of disease incidence (the response variable *y_ijkl_*) was defined as the number of disease cases per county for ASD (*k* = 1) and ID (*k* = 2). Subscripts *i* and *ij* were used to indicate a state and a county nested within that state, and subscript *l* to indicate gender. The second assumption was that the logarithm of Poisson rate (λ*_ijkl_*) was expressed as a linear combination of fixed and random effects.
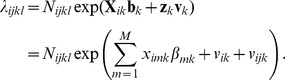
Here matrix **X** *_k_* is the design matrix for the fixed effects associated with disease *k*; **b** *_k_* is the corresponding vector of unknown regression weights; **z** *_k_* is a design matrix for random effects; **v** *_k_* is the vector of random effects; *v_ik_*, *v_ijk_*, are *i*-state- and *ij*-county-specific random effects for disease *k*. The fixed-effect design matrix is simply a matrix of county-specific zero-centered properties, such as the mean income, or proportions of ethnic groups. The design matrix **z** has a very simple form: entries of 1 for random effects of a given county and corresponding state, and zeros in all other cells. *N_ijkl_* is a state-, county-, disease- and gender-specific offset—the total number of people with a specified gender living within a given county.

The third assumption was that data was hierarchical: the zero-centered random effects were independently introduced at *i*-state and *ij*-county levels. However, the random effects associated with the two diseases (1 and 2) were geographically correlated,
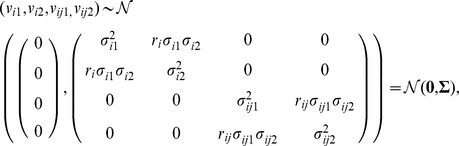
where *σ* ^2^ *_ik_*, and *σ* ^2^ *_ijk_*, are variances of state-level and county-level random effects for disease *k* and *r_i_*, and *r_ij_* are correlation coefficients for random effects for diseases 1 and 2 at the state and county levels, correspondingly.

Together these assumptions define the following likelihood for the given *ij*-county, *i*-state, *l*-gender and *k*-disease:
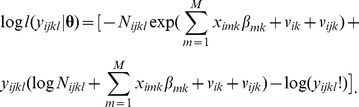
The full log-likelihood is obtained by summing the individual log-likelihoods specific to each *y_ijkl_* over all possible indices.

While estimates varied slightly across different implementations of the model and estimation approaches, the major trends were identical across all. Here we present the results of the Markov chain Monte Carlo/Empirical Bayes analysis. The estimation methods and starting parameter values varied considerably across the implementations. For example, SuperMix started with finding an analytical solution of the fixed-effect part of the equations and then estimated parameters for the full model involving random effects. The Markov chain Monte Carlo-based GLLAMM started with a random set of parameter guesses and then rather quickly discovered the high-probability area of parameter values.

### Confounders

We tested several putative confounding variables, such as county-specific median mother's age at childbirth, and the proportion of county population in the childbearing age. While these variables produced statistically significant fixed-effect coefficients, they did not affect the relationship between the outcome variable and the compound environmental predictor variables as would be expected if these added variables were true confounders.
